# Two Types of Refutation in Philosophical Argumentation

**DOI:** 10.1007/s10503-022-09583-5

**Published:** 2022-09-12

**Authors:** Catarina Dutilh Novaes

**Affiliations:** 1 Vrije Universiteit Amsterdam, Amsterdam, The Netherlands; 2 University of St. Andrews, St. Andrews, United Kingdom

**Keywords:** *Elenchus*, Socrates, Aristotle, Gettier problem, Lakatos, Counterexamples

## Abstract

In this paper, I highlight the significance of practices of *refutation* in philosophical inquiry, that is, practices of showing that a claim, person or theory is wrong. I present and contrast two prominent approaches to philosophical refutation: refutation in ancient Greek dialectic (*elenchus*), in its Socratic variant as described in Plato’s dialogues, and as described in Aristotle’s logical texts; and the practice of providing counterexamples to putative definitions familiar from twentieth century analytic philosophy, focusing on the so-called Gettier problem. Moreover, I discuss Lakatos’ method of proofs and refutations, as it offers insightful observations on the dynamics between arguments, refutations, and counterexamples. Overall, I argue that dialectic, in particular in its Socratic variant, is especially suitable for the philosophical purpose of questioning the obvious, as it invites reflection on one’s own doxastic commitments and on the tensions and inconsistencies within one’s set of beliefs. By contrast, the counterexample-based approach to philosophical refutation can give rise to philosophical theorizing that is overly focused on hairsplitting disputes, thus becoming alienated from the relevant human experiences. Insofar as philosophical inquiry treads the fine line between questioning the obvious while still seeking to say something significant about human experiences, perhaps a certain amount of what Lakatos describes as ‘monster-barring’—a rejection of overly fanciful, artificial putative counterexamples—has its place in philosophical argumentation.

## Introduction

Arguments and argumentation occupy a central role in philosophical practice, both historically and currently; indeed, one may even go as far as saying that ‘giving and asking for reasons’ in favor or against different positions is at the very heart of philosophy. (Which is compatible with recognizing that there are also non-argumentative elements in philosophical practice.) Is there something distinctive about philosophical argumentation when compared to argumentative practices in other domains?^1^ These other domains include politics, the law, scientific fields, and instances of argumentation in everyday life—for example, deciding on travel plans among friends. On the one hand, it seems plausible that philosophical argumentation will draw on more general, all-purpose socio-cognitive skills and practices pertaining to arguments. On the other hand, philosophical arguments may well differ substantially from more mundane communicative practices. Philosophical inquiry consists in questioning the obvious, that is, in revealing that what appears to be ordinary and uncomplicated is in fact not so straightforward (Dutilh Novaes 2019). If this is the case, then one might expect that, while bearing similarities with argumentation elsewhere, philosophical inquiry will also rely on specific argumentative practices suitable for the overall goal of questioning the obvious (which is quite different from, for example, achieving consensus in a coordination problem).

What are then the features specific to philosophical argumentation (if any)? In this paper, I highlight the significance of practices of *refutation* in philosophical inquiry, that is, practices of showing that a claim, person or theory is wrong (in a suitable sense of ‘wrong’). (Etymologically, to refute means ‘to strike back’.) In most other settings, dialogical communication tends to be largely cooperative (Ginzburg 2016) and to follow norms of credulity (Aikin 2011). Requests for (additional) reasons and justifications to support one’s claims are often perceived as rude and confrontational, indicating suspicion of insincerity. In philosophical argumentation, by contrast, the critical stance of pressing for further reasons and finding fault in arguments is perfectly acceptable practice. In particular, the use of *counterexamples*, no matter how far-fetched and artificial they may be, is one prominent strategy for philosophical refutation (at least in some traditions such as analytic philosophy, but also in e.g., medieval scholasticism). In everyday life and in most other specific domains, a position or idea will be viewed as ‘good enough’ if it applies to a wide enough range of the relevant cases, even if it fails to apply to some limit cases. By contrast, philosophical argumentation tends to have a much lower tolerance for exceptions.

To be clear, the claim here is not that the central role occupied by refutations is unique to philosophy. In legal contexts, for example, especially in adversarial justice systems, the goal is often to show the untenability of the positions of one’s opponent. Similarly, in mathematics, proofs are put under rigorous scrutiny and are refuted when mistakes are found (as described by Lakatos in *Proofs and Refutations*, to be discussed below). The claim to be defended here is rather that refutations are crucial for the philosophical goal of ‘questioning the obvious’. Indeed, while in most scientific disciplines, empirical testing is the quintessential way to criticize or disprove scientific claims (Strevens 2021), in philosophy this functional task is primarily fulfilled by argumentative refutations.^2^

In what follows, I present and contrast two prominent approaches to philosophical refutation: ancient dialectic as described in Plato’s dialogues and Aristotle’s logical texts, and the practice of providing counterexamples to putative definitions familiar from twentieth century analytic philosophy (which is still widespread today, though perhaps less so than previously). One could describe them as two species of the genus ‘philosophical refutation’. The main difference between them is that, while in ancient dialectic it is primarily a *person* who is refuted—shown to hold an incoherent set of beliefs—in analytic philosophy refutation aims primarily at claims and definitions. While it might seem that the second approach is preferrable, as it is less *ad hominem*, I will argue that it may give rise to philosophical theorizing that is overly focused on hairsplitting disputes, thus becoming alienated from the relevant human experiences. By contrast, dialectic, in particular in its Socratic variant, is especially suitable for the philosophical purpose of questioning the obvious, as it invites reflection on one’s own doxastic commitments and on the tensions and inconsistencies within one’s set of beliefs.

The paper proceeds as follows. Section 2 discusses refutation in ancient Greek dialectic, in its Socratic and Aristotelian versions. Section 3 discusses the twentieth century debate on the analysis of knowledge as it developed in the aftermath of Gettier’s influential critique of the JTB concept of knowledge. Section 4 turns to Lakatos’ account of proofs and refutations in mathematics, which contains observations about different reactions to refutations and counterexamples that are also applicable to philosophical argumentation. To illustrate their philosophical relevance, Lakatos’ insights are applied to the specific example of the Gettier problem. Section 5 offers some concluding remarks. Throughout the paper, it is argued that, on the whole, dialectic (Socratic dialectic in particular) offers a more fruitful approach to philosophical refutation than analytic philosophy’s focus on the formulation of counterexamples.

## Refutation in Ancient Dialectic^3^

In ancient Greek philosophy, two influential modes of philosophical argumentation were the long speeches associated with the sophists/rhetoricians (presumably modeled on debating procedures in the assembly and in a court of law), and dialogical interactions where speakers took turns in quick succession. These dialogues came to be known as the practice of *dialectic*, which consists in conversations following a specific, fairly systematic structure: verbal bouts between two interlocutors, a questioner and an answerer, in front of an audience, possibly with a referee or judge. The art of dialectic seems to predate Socrates and Plato, understood in its literal sense of ‘*dialektikê*’, ‘the art of conversing’.^4^ Some sources attribute skills of question-and-answer bouts to the sophists themselves (Nehamas 1990) and the philosophers of the Eleatic school (Parmenides, Zeno) may also have engaged in such dialogues.

Dialecticians maintained that the dialogical form was more conducive to truthful speech than long speeches because questioner and answerer can keep each other in check, as it were. A passage from *Republic* VII illustrates this point (transl. Grube, emphasis added):

The man who cannot by reason distinguish the Form of the Good from all others, who does not, *as in a battle, survive all refutations*, eager to argue according to reality and not according to opinion, and who does not come through all the tests without faltering in reasoned discourse—such a man you will say does not know the Good itself, nor any kind of good.^5^ (534b-c)

Moreover, for pedagogical purposes, short speeches are more conducive to enhancing the pupils’ understanding, as they are required to engage with each step along the way (e.g., the famous passage from the *Meno* where Socrates shows the slave boy how to double the area of a square). Dialectical encounters thus allow for a careful scrutiny of views, and it is the presupposition of a certain level of adversariality between questioner and arguer, or at least a highly critical stance, that ensures the truth-conduciveness of dialectic, as suggested in the passage from the *Republic* just quoted (‘as in a battle’).

As the passage above also indicates, a key component of dialectic thus understood is the concept of *refutation*, or *elenchus* in Greek: questioner aims at refutation, answerer tries to avoid being refuted. But what exactly is an *elenchus*? Readers of Plato will undoubtedly recall the numerous instances where Socrates, by means of questions, elicits a number of discursive commitments from his interlocutors, only to go on to show that, taken together, these commitments are incoherent.^6^

A Socratic *elenchus* can be understood as a test of the overall coherence of a person’s beliefs: “a test that shames those who fail it, and which cleanses their soul through that shaming” (Castelnérac and Marion 2009, p. 51). The etymology of *elenchus* is indeed related to shaming (Lesher 2002), and at least in some cases it seems that Socrates is out to shame the interlocutor by exposing the incoherence of their set of beliefs, which suggests a hostile stance. However, as noted by Socrates himself in the *Gorgias* (470c7-10), refuting is also what friends do to each other, a process whereby someone rids a friend of mistaken beliefs. (Similarly, an *elenchus* can also have pedagogical purposes; in some dialogues, Socrates clearly adopts the stance of a teacher.) Indeed, in the ancient Greek context, shame did not have the negative connotation that it presently has; it is best understood in the sense of virtues such as *honesty* and *humility*.

To explain Socratic dialectic, Plato introduces two metaphors alluding to physiological processes, and to practitioners who promote the good health and well-being of those in need (women in labor, sick individuals): Socrates as the midwife in the *Theaetetus* (Sedley 2004), and Socrates as the physician in the *Gorgias* (Moss 2007). Importantly, just as with physical labor, giving birth to truths can be a painful, difficult experience.

In the *Gorgias*, Plato elaborates on the analogy between tending to the needs of the body and tending to the needs of the soul. What is at stake is the confrontation between two conceptions of argumentation: the one defended and practiced by rhetoricians such as Gorgias, and the one defended and practiced by Socrates, the philosopher/dialectician. The former focuses on domination of others as a means to secure personal advantage (at least according to Plato/Socrates), while the latter approaches argumentation as a mutually beneficial activity that follows naturally from the pursuit of truth and wisdom (Irani 2017). In particular, in this dialogue Socrates attempts (unsuccessfully, it seems) to convince his interlocutors to choose the path of justice and to lead a just life. If philosophical *logoi* can persuade those tempted by more frivolous ways of life to choose justice, then it will have had a therapeutic effect for the soul, comparable to what a medical doctor can achieve by curing the ailments of the body.

Plato/Socrates compares the rhetorician to a pastry chef, who offers delicious but unhealthy treats to the body. By contrast, the philosopher is like a true physician, who restores the health of a sick person even if the treatment itself is rather unpleasant. Philosophy is a beneficial craft, whereas rhetoric (as pastry-baking) is nothing but a flattering knack. And yet, between the doctor and the pastry chef, the ignorant will oftentimes choose the pastry chef: the flattering knacks of pastry-baking and rhetoric are far better at *persuading* most people than medicine and Socratic dialectic (Moss 2007).

But what exactly is the benefit that the ‘physician of the soul’ Socrates can confer to his ‘patients’ by means of sustained questioning? The goal is to cleanse the troubled soul from false beliefs (a theme also found in the *Sophist*), which is achieved by an *elenchus*. By asking questions and eliciting discursive commitments, Socrates exposes internal inconsistencies in a person’s set of beliefs, thus prompting a re-evaluation thereof. The twist here is that he who is refuted is in fact the one who most benefits from the dialectical interaction:

And what kind of man am I? One of those who would be pleased to be refuted if I say something untrue, and pleased to refute if someone were to say something untrue, yet not at all less pleased to be refuted than to refute. For I think that being refuted is a greater good, in so far as it is a greater good for a man to get rid of the greatest evil himself than to rid someone else of it—for I think there is no evil for a man as great as a false belief about the things which our discussion is about now [justice and the good life]. (*Gorgias* 458a2-b1)

Note that ‘sticking to one’s guns’ (not changing one’s mind) is typically viewed as constituting victory in a game of argumentation, while being shown to be mistaken—being refuted—would correspond to a defeat (Cohen 1995). Here Socrates turns these ‘winning/losing conditions’ around and proclaims that it is better to be refuted than to refute, as to be refuted will entail a genuine improvement to the soul’s health. It is specifically in this sense that engaging in argumentation can have a therapeutic effect, despite the initial discomfort involved in being proved wrong.

Thus understood, a Socratic *elenchus* is a quintessential method to ‘question the obvious’, to show that one’s commonsensical belief system contains multitudes of tensions and contradictions. But as is well known, going around questioning the obvious and ridding people of false beliefs did not make Socrates particularly popular; he described himself as “the gadfly of the Athenian people” (in the *Apology*), and his defying attitude eventually cost him his life.

The therapeutic approach to refutation is quite specific to Socrates, and is absent for example in Aristotle’s regimented account of dialectic. The *Topics* and its ‘ninth chapter’, the *Sophistical Refutations*, offer a regimentation and systematization of practices hitherto dictated by tacit rather than explicit rules. Aristotle’s texts provide a general description of these practices which can be summarized thus:

First of all, there are the agents: the questioner and the answerer. There may also have been an audience (*Sophistical Refutations* 16 175a20–30). The questioner has two main jobs: first, to extract a thesis, the ‘starting point’ for the debate from the answerer; second, to try to force the answerer to admit the contradictory of that starting point, by getting the answerer to agree to certain premises. Alternatively, the questioner can try to reduce the thesis to absurdity. In either case, the questioner aims to refute the answerer. Crucially, the starting point should be something that can be affirmed or denied (*Topics* 8.2. 158a14–22). For example, ‘what is knowledge?’ would not be allowed as a starting point, as the answerer cannot reply ‘yes’ or ‘no’. The answerer, on the other hand, has only one task, which is to remain un-refuted within a fixed time (*Topics* 8.10. 161a1–15). If the answerer is refuted, then the answer should make clear that it is not their fault, but is due solely to the starting point (*Topics* 8.4. 159a18–22). (Duncombe & Dutilh Novaes, 2016, p. 3)

Notice here the (seemingly) *ad hominem* character of these dialogues: questioner wants to refute answerer (or answerer’s position), whereas answerer tries to defend herself by maintaining the consistency of her discursive commitments (Castelnérac and Marion 2009), thus avoiding refutation. They aim for different goals, and are ‘adversaries’ in the sense that these different goals cannot simultaneously obtain (Dutilh Novaes 2021).

However, Aristotle also stresses the *cooperative* dimension of dialectic. In Chapter 4 of Book VIII of the *Topics*, Aristotle distinguishes three modes of engaging in argumentation: eristic, which is purely competitive; didactic, for teachers and learners; and dialectic, for inquiry. He then claims that the details of the third mode have never been properly described, which he then sets out to do.

But when it comes to dialectical meetings among people who engage in arguments not for the sake of competition, but for testing and inquiry (*peiras kai skepseos*), it has never been spelt out what the answerer must aim at, or what sorts of things he must grant and what not in order to <count as> defending his thesis well or not. (*Topics* 159a33-37)

The key notions here are ‘testing and inquiry’. Testing echoes the notion of *elenchus* as testing described above; inquiry, in turn, seems to be “a means for exploring the consequences of different opinions as a part of philosophical inquiry” (Smith 1997, p. 129) Inquiry in particular is a cooperative endeavor at a higher level, as there is a ‘common work’ to be accomplished. However, when one of the interlocutors is not sufficiently cooperative, then the dialogue becomes purely adversarial:

For it is not in the power of one participant alone to see that their common work is well accomplished. There are times, then, when it is necessary to attack the speaker, not the thesis—when the answerer is particularly abusive and ready to pounce on the questioner with the contrary of whatever he asks for. By being cantankerous, then, these people make discussions competitive and not dialectical. (*Topics* 161a20-25)

And since it is a poor participant who impedes the common work, so it is clearly also in an argument. For there is also a common project in these (except for competitive ones: in these, it is not possible for both to achieve the same goal, for it is impossible for more than one to win.) (*Topics* 161a37-161b1)

Dialectic as understood by Aristotle is thus characterized by a complex interplay between adversariality and cooperation. While at a lower level the practice appears purely competitive (questioner seeks to refute answerer; answerer seeks to avoid being refuted), at a higher level, participants are in fact also cooperating towards a common goal. An important difference with respect to Socratic *elenchus* is that, in an Aristotelian dialectical encounter, one specific thesis is singled out, and then questioner tries to get answerer to conclude the contradictory of the initial thesis (with auxiliary premises granted along the way). A Socratic *elenchus* is more holistic in that it merely shows that the various discursive commitments of answerer, taken together, are incoherent, without singling out any one of them specifically as problematic (Matthews 2009; Dutilh Novaes 2016).

In both cases, however, a refutation is a powerful philosophical tool in that it serves to refine and improve one’s views and positions by means of critical scrutiny. A refutation seems to work best in a multi-agent setting where interlocutors examine each other’s views critically (a point that was to be emphasized millennia later by John Stuart Mill (Mill 1999)). In these situations, a refutation may appear to be a ‘personal attack’ but may in fact lead to significant epistemic improvement; either through a reevaluation of one’s beliefs (in Socratic dialectic) or through an exploration of what follows from different discursive commitments (in Aristotelian dialectic). Its apparent confrontational nature is tempered by a therapeutic component in Socratic dialectic, and by a cooperative component in Aristotelian dialectic.^7^

## The Analysis of Knowledge in the Second Half of the 20th Century

An influential philosophical debate in the second half of the twentieth century offers another significant illustration of philosophical refutation: the debate on the *analysis of knowledge* sparked by Gettier’s (1963) paper ‘Is justified true belief knowledge?’ Naturally, the project of analysing knowledge itself has a long history. Analysis, understood as the decomposition of a concept into its constituent parts and the formulation of definitions, has been a common feature of philosophical practice for millennia. Indeed, questions of the form ‘What is X?’, where X is a philosophically significant concept, are the starting point for many of Plato’s dialogues (Matthews 2009). One of Plato’s most celebrated dialogues, the *Theaetetus*, is concerned precisely with the question of how best to understand and define *knowledge* (Chappell 2019). The character Theaetetus, a brilliant young mathematician, proposes three definitions of knowledge: knowledge as perception, knowledge as true belief, and knowledge as true belief with an ‘account’ (*logos*). In turn, three proposals on how to understand *logos* in this context are discussed. All three main proposals, and all three specifications of *logos*, are shown by Socrates not to resist refutation and are therefore rejected. The *Theaetetus* is thus an aporetic dialogue: at the end of the dialogue, we still do not know what knowledge *is*. We only come to identify three things that knowledge is *not*.

In the twentieth century, analysis was explicitly placed at the core of philosophical practices by so-called *analytic* philosophers. Analytic philosophy arose from the rejection of British Idealism by G.E. Moore and Bertrand Russell at the turn of the nineteenth to twentieth century (Beaney 2017). While united by a common enemy, these two founding figures in fact held different conceptions of philosophical analysis: for Moore, philosophical inquiry was expected to remain closely aligned with commonsensical beliefs, whereas Russell espoused a more revisionary conception of analysis, wherein philosophical conclusions might well clash with commonsensical beliefs (e.g., in his analysis of the semantics of definite descriptions) (Dutilh Novaes and Geerdink 2017).

Moreover, Russell’s conception of analysis relied extensively on formal logical tools that had been recently developed by Frege and others in their work on the foundations of mathematics. Russell thus inaugurated the ‘scientific philosophy’ approach, which was to influence greatly the Vienna Circle thinkers. They in turn significantly shaped the development of philosophy in the twentieth century, especially in the USA, where many of them emigrated to before or during WWII. As a result, philosophical argumentation in this tradition came to be importantly influenced by mathematical ways of arguing, in particular the prominent position accorded to *counterexamples*.^8^ Gettier himself belonged very much to this tradition,^9^ having written his dissertation on Russell’s theory of belief. At the same time, the Moorean emphasis on common sense later developed into the centrality of intuitions in philosophical inquiry (more on which below) (Dutilh Novaes and Geerdink 2017).

It is against this background that analytic epistemologists in the second half of the twentieth century spent a great deal of time involved in the project of providing a suitable analysis of knowledge. The twentieth century debate on the nature of knowledge starts, in Gettier’s influential 1963 paper, with an argument about what knowledge is *not* (thus echoing the *Theaetetus*), namely justified true belief. The JTB analysis, as it became known, was presented by its detractors as the ‘classic’ conception of knowledge, which was allegedly then refuted by Gettier’s counterexamples.^10^ More precisely, while each element of JTB may be *necessary* for knowledge (many epistemologists still hold views in this vicinity), Gettier’s argument purports to show that they are collectively not *sufficient*. This means that at least some cases of justified true belief will not count as knowledge (such as Gettier cases), thus giving rise to a formidable cottage industry of (presumed) instances of justified true beliefs that should apparently not be considered as instances of knowledge (in the sense that they do not ‘intuitively’ count as knowledge—see methodological remarks below). Importantly, while in ancient Greek dialectic the focus of a refutation lay in getting one’s interlocutor to grant a contradiction or some other embarrassing outcome (which may but need not include recourse to counterexamples), in twentieth century analytic philosophy (and beyond), a philosophical refutation relies primarily on the formulation of *counterexamples* (to claims, definitions etc.) (Williamson 2005).

Gettier (Gettier 1963) presents two cases where a true belief is inferred from a justified false belief, and notes that these true beliefs cannot properly count as knowledge, despite (arguably) being justified true beliefs. In one example, two men, Jones and Smith, are being considered for a job. Smith believes that Jones will get the job, and furthermore he believes (having counted them) that Jones has 10 coins in his pocket. Hence, Smith holds the belief that the man who will get the job has 10 coins in his pocket, as this is a logical consequence of his other two beliefs. However, unbeknownst to him, Smith himself will get the job, and moreover he also happens to have 10 coins in his pocket. Thus, his belief that the man who will get the job has 10 coins in his pocket is *justified* thanks to the principle of epistemic closure: he has solid albeit mistaken reasons to think that Jones will get the job, he counted the coins in Jones’ pocket, and these two justified beliefs entail the ‘Gettierized’ belief. And this belief is also *true*—as it happens, the man who will in fact get the job, namely Smith, does have 10 coins in his pocket. But there is no substantive connection between these two facts, namely the justifiedness and the truth of this belief; it is a matter of *sheer luck* that Smith ends up with this justified true belief, and for this reason Gettier claims that it cannot reasonably be viewed as an instance of knowledge. Indeed, the idea that knowledge is incompatible with (epistemic) luck is a frequent observation in much of these debates, historical as well as recent ones (Pritchard 2005).

In light of Gettier cases, which appear to refute the JTB analysis, many epistemologists embarked on the project of suitably repairing JTB. Initially, the two main strategies pursued in the literature were: attempts to strengthen the justification condition so as to rule out Gettier cases as proper cases of *justified* belief; and attempts to amend JTB with an appropriate fourth condition. These latter attempts are schematically referred to as JTB + *X* accounts, where X stands for the fourth condition which should distinguish Gettier cases, where luck is involved, from ‘real’ instances of knowledge. The ensuing debate is aptly summarized thus:

Gettier’s paper launched a flurry of philosophical activity by epistemologists attempting to revise the JTB theory, usually by adding one or more conditions, to close the gap between knowledge and justified true belief. […] When intuitive counterexamples were proposed to each theory, epistemologists often responded by amending their theories, complicating the existing conditions or adding new ones. (Ichikawa & Steup, 2018) (Section 7)

Various iterations of these epicycles ensued, yielding increasingly convoluted new analyses of knowledge, which in turn gave rise to new, often far-fetched counterexamples to the various proposals (Gendler and Hawthorne 2005). One particularly fanciful scenario is the well-known Barn County case (Goldman 1976). Barn County is a (fictional, of course) county with a very peculiar feature, namely that the main road cutting across the county contains several barn-facades, which are structures that look exactly like barns from the road but are not barns. Henry is driving through the county, and upon seeing the many barn-facades, he justifiedly believes of each of them that it is a real barn (his perceptual organs are perfectly functional, and he has no reason to suspect deception); these are justified but false beliefs. He then sees the one and only real barn in the county, and here again concludes that it must be a barn (as with the barn-facades). Only this time, his belief happens to be true, and it is also justified (as are his false beliefs about the barn-facades being actual barns). And yet, since it is also a matter of luck that, *this time*, he is looking at an actual barn, this justified true belief does not count as knowledge. Barn County is viewed as a counterexample to a number of revised JTB-like analyses, in particular those based on safety conditions, reliabilism, and virtue-theoretic approaches (Ichikawa and Steup 2018).

Indeed, it seems that Gettier-like cases will arise no matter how cleverly one amends one’s analysis of knowledge along the JTB lines. Zagzebski (Zagzebski 1994) argued in an influential paper that no analysis sufficiently similar to JTB, with an additional condition X, could ever avoid the problems highlighted by Gettier cases. Indeed, she presents a ‘recipe’ to construct Gettier cases: start with an example of a case where a subject has a justified false belief that also meets condition X, and then modify the case so that the belief is true merely by luck. A natural reaction to Zagzebski’s argument is to accept the overall failure of the project, and to conclude that knowledge is in fact unanalyzable. It is this sentiment that motivates for example the ‘knowledge first’ research program (Williamson 2002). Another popular response is to focus specifically on what is arguably the core of the problem, namely the occurrence of luck, and thus to develop suitable anti-luck conditions (as suggested by Zagzebski (1994) herself). As a result, epistemic luck has since become a lively topic of investigation and discussion among epistemologists (Pritchard 2005).

Looking back on this decades-long debate, some tentative conclusions present themselves. Along the way, it has sparked a number of important developments (e.g., virtue epistemology and investigations on the notion of epistemic luck). But it fully absorbed generations of epistemologists who might also have spent some of their time and attention on other relevant, interesting or fruitful topics. There was already something odd about the original Gettier cases, but as the debates progressed, the concocted scenarios intended to serve as counterexamples to the revised proposals became even more distant from realistic situations. (Fake barn facades, really?) Do these extremely implausible scenarios still tell us something about notions of knowledge that are relevant to human experiences?

Relatedly, how reliable are our *intuitions* about what should or should not count as knowledge in these far-fetched scenarios? Most of these debates proceeded on the assumption that these intuitions should be the metric to evaluate the extensional adequacy of proposed analyses of knowledge. Now, even assuming that they might be reliable in realistic situations (which is not a given),^11^ the artificiality of the counterexamples seems to pose additional challenges to this method. Finally, notice the expectation that a proposed analysis of knowledge would get the extension of the concept *exactly right*, with no exceptions allowed. Arguably, in other, more mundane argumentative contexts, the demand that there be absolutely no counterexamples to a claim is quite unnatural; far-fetched, unlikely scenarios are typically viewed as not constituting knock-out objections to otherwise sensible claims. This is attested for example by the pervasiveness of generic generalizations, which resist extreme counterexamples (Leslie and Lerner 2016).

Thus, while it is to be expected that philosophical inquiry will question the obvious and defy common sense (Dutilh Novaes 2019), in particular by means of refutations and counterexamples, the Gettier problem and the ensuing debates show that philosophical argumentation of this kind can also become alienated from human experiences. Especially the demand that definitions and claims be immune to every conceivable counterexample, no matter how far-fetched and unrealistic, seems to pave the way for epicycles of increasingly convoluted arguments, refutations, and counterexamples. This is consistent with the view that philosophy should defy common sense, but this approach can also lead to hairsplitting disputes over overly abstract, ethereal issues by means of fanciful examples and strange thought experiments.^12^

## Lakatos on Proofs and Refutations

In view of these potential issues with the counterexample-based approach to philosophical refutation, a more systematic reflection on the formulation and responses to counterexamples seems to be called for. As it happens, one of the most interesting analyses of the dynamics between arguments, refutations, and counterexamples is to be found in a text that purports to discuss the dynamics of argumentation in *mathematics*: Lakatos’ *Proofs and Refutations* (Lakatos 1976). True enough, Lakatos’ model is inspired by philosophical ideas, in particular Hegelian dialectic (Larvor 2001), so it this sense it is not surprising that the model is also applicable to philosophical argumentation. In fact, whether the model as a whole offers a suitable account of mathematics, either descriptively or normatively, is a moot point.^13^ But it offers an insightful general reflection on the dynamics of refutations and counterexamples in argumentation which is also relevant for philosophical argumentation, in particular in sub-areas of philosophy that have been significantly influenced by mathematical reasoning (such as the ‘scientific’ strand within analytic philosophy).

Before we turn to Lakatos specifically, it seems fitting to distinguish between different types of counterexamples in terms of what they are counterexamples *to*. Gettier cases were presented as counterexamples to the analysis of knowledge as justified true belief, which posits a purported equivalence between two classes of phenomena, namely ‘knowledge’ and ‘justified true belief’. Thus, a counterexample can be formulated to an analysis of a concept X in terms of its putative constituents ABC. But counterexamples can also pertain to statements, for example to universal statements (‘All ravens are black’ is refuted by the existence of a white raven) or conditional statements (‘If there is smoke, then there is fire’ is refuted by an instance of smoke arising in the absence of fire). Furthermore, deductive arguments and their inferential steps can also be refuted by counterexamples, which are situations where the premises are the case but the conclusion is not (thus showing that the argument or step is not necessarily truth-preserving after all). Since his focus is on mathematical proofs, which are deductive arguments *par excellence*, Lakatos is primarily interested in the latter type of counterexamples. However, his remarks on the different reactions to counterexamples, to be discussed below, seem to apply (mutatis mutandis) to other types of counterexamples as well.

The main text of *Proofs and Refutation*s consists in a classroom dialogue between a teacher and students named after letters of the Greek alphabet, discussing various (attempted) proofs of Euler’s conjecture for polyhedra. The conjecture states that the vertices (V), edges (E) and faces (F) of a polyhedron satisfy the formula *V* − *E* + *F* = *2*. The dialogue is presented as a rational reconstruction of the actual historical development of (attempted) proofs for the conjecture and their refutations; the different students are portrayed as representing the various positions and reactions.^14^

The dialogue starts with the teacher presenting a proof (due to the nineteenth century mathematician Cauchy) supporting the conjecture, which the students then go on to scrutinize and criticize for various reasons. At each objection, the proof is modified so as to withstand the force of the objection, for example by restricting the relevant definition so that the counterexample proposed is now excluded from the domain of applicability of the conjecture. Through this process, it becomes clear that many of the key concepts involved (e.g., the concept of a polyhedron itself) were in fact vague and poorly understood at the starting point, and through the dialectic of proofs and refutations these concepts become sharpened and clarified (Tanswell 2017) (section 3.3).

Crucially, this process is open-ended. Lakatos maintains that there is no end-point for a mathematical proof: a proof remains open to refutation even when it comes to be accepted as correct by the relevant ‘certifiers’ at a given point in time. Indeed, the same applies to the mathematical concepts involved: they continue to develop overtime, as open-textured concepts (Tanswell 2108). This is, of course, also true of philosophical concepts: they continue to evolve and are constantly reshaped and refined, in no small measure thanks to the dynamics of critical evaluation, refutation, and counterexamples.

Throughout the dialogue, a typology of responses to putative counterexamples and problematic cases is presented. To illustrate the relevance of these types of responses to philosophical debates, in what follows I draw systematic comparisons between the Euler conjecture and the JTB analysis, where polyhedra are analogous to knowledge (the analysandum) and the conjecture in terms of V, E and F corresponds to the JTB analysis. The analogy is imperfect: the problem with the JTB analysis is that it allegedly overgenerates—some instances of justified true beliefs are seen as not counting as proper instances of knowledge—whereas Euler’s conjecture does not claim that every solid that satisfies the formula *V* − *E* + *F* = 2 is a polyhedron. Rather, it claims that every polyhedron satisfies the formula in question (whereas for the JTB analysis the implication should run in both directions: K iff JTB). Still, this dissimilarity should not affect the usefulness of the analogy for our purposes.

First, there is the *surrender response*, thus described by one of the students, Gamma: “A single counterexample refutes the conjecture as effectively as ten. The conjecture and its proof have completely misfired. Hands up! You have to surrender.” (Lakatos 1976, p. 13). This happens when a single counterexample is viewed as having such a devastating effect that the claim in question is simply abandoned. With respect to the analysis of knowledge, this would amount to completely abandoning the JTB analysis in view of Gettier cases. As we know, this is not what happened initially: while the Gettier cases were viewed as revealing a serious limitation in the naïve JTB analysis, the main response was to try to repair JTB, either by strengthening the notion of justification or by adding a suitable fourth component X to JTB. However, later on at least some epistemologists viewed Zagzebski’s ‘recipe’ to build Gettier cases as reason enough to abandon the JTB approach altogether, and thus may be said to have ‘surrendered’ (not to a single counterexample but to an argument showing that new counterexamples can be continuously generated).

Next, there is the *monster-barring response*, thus described by Delta: “It is the ‘criticism’ that should retreat. It is a fake criticism. The pair of nested cubes is not a polyhedron at all. It is a monster, a pathological case, not a counterexample.” (Lakatos 1976, p. 14). The point here is that the purported counterexample, a solid that does not satisfy the formula *V *− *E* + *F* = *2*, is in fact not a legitimate counterexample because it is not a polyhedron in the first place: it is a monster. Thus, it should not count as a valid refutation of the initial claim. In JTB terms, a monster-barring response might be to contend that Gettier cases do not really constitute instances of justified true belief; this is the reaction of those who seek to formulate a stricter notion of justification such that Gettier cases would fail the justification requirement of JTB (such as Chisholm 1977). Moreover, the objection that the Barn County case is too much of an oddity to tell us anything about more naturalistic conceptions of knowledge (a point that I myself find rather compelling) may also be viewed as belonging to the monster-barring family, insofar as it questions the legitimacy of the purported counterexample.

A cousin of the monster-barring response is the *exception-barring response*: no conjecture or claim is generally valid, but only valid in a certain domain that excludes exceptions. The challenge then becomes that of characterizing precisely the domain in question. (For Euler’s conjecture, we now know that it applies to all polyhedra except for those with holes running through them (Kirk 2020).) In the JTB case, what would be required is an *independent* characterization of knowledge, one which rules out Gettier cases as instances of knowledge. However, formulating such a characterization is precisely what the JTB analysis attempted (and failed) to do. Instead, in the JTB debates, the metric used to establish the relevant domain, namely the concept of knowledge in its intension and extension, was rather imperfect: whether a certain instance ‘intuitively’ appeared to count as knowledge. The problem with exception-barring is that it may become the ad hoc exercise of constantly formulating additional clauses as new counterexamples come along, leading precisely to the somewhat frustrating epicycles observed in the JTB literature.

Finally, there is the *lemma-incorporation response*. The general thought is to take a good look at what went wrong in the original proof, such that a counterexample emerged, and to address the problem by incorporating additional clauses to the formulation of the conjecture. While there may still be something a bit ad hoc about lemma incorporation (it is prompted by counterexamples that happen to be formulated), it should in fact lead to a more thorough examination of the argument as a whole and how to repair it. There seems to be a neat analogy here with the JTB debates: as they progressed, it became clear that the underlying phenomenon in Gettier cases was that of *epistemic luck*. The realization of the connection between knowledge and (the absence of) luck and the subsequent focus on anti-luck conditions may be viewed as a successful instance of ‘lemma incorporation’.

After running through these various responses to problematic cases, the general method of proofs and refutations is finally described by means of four rules (Lakatos 1976) (pp. 50 and 58). Rule 2 seems particularly relevant for philosophical inquiry:

Rule 2. If you have a global counterexample discard your conjecture, add to your proof-analysis a suitable lemma that will be refuted by the counterexample, and replace the discarded conjecture by an improved one that incorporates that lemma as a condition. Do not allow a refutation to be dismissed as a monster. Try to make all hidden lemmas explicit. (Lakatos, 1976) (p. 50)

With a few suitable adaptations, Lakatos’ rules for the method of proofs and refutations seems to provide sensible guidance also for philosophical inquiry. Indeed, a philosophical refutation should typically lead to an improved position, at the very least as it exposes inconsistencies and other inadequacies. Of course, insofar as a refutation has primarily a *negative* epistemic import—it is aimed at exposing errors and shortcomings—it needs to be complemented with strategies to produce new positive claims (lest one ends up becoming a full-blown skeptic). Lakatos’ Rule 2 describes how a new substantive claim (conjecture) emerges from a refutation, and the gist of it seems to be equally applicable to philosophical inquiry: try to make presuppositions explicit, take refutations and counterexamples seriously (do not dismiss them easily as irrelevant ‘monsters’), improve and refine new conjectures by means of refutations. Nevertheless, the advice not to easily dismiss counterexamples as monsters may well be more reasonable in mathematics, where the topic of investigation are abstract entities and structures, than in philosophy. Insofar as philosophical inquiry treads the fine line between questioning the obvious while still seeking to say something significant about human experiences, perhaps a certain amount of ‘monster-barring’ in philosophical argumentation has its place.

## Conclusions

The main claim defended in this paper is that, if one accepts the view that philosophical inquiry consists primarily in questioning the obvious, refutation will be an essential component of philosophical argumentation. I illustrated this claim by discussing two ways of engaging in philosophical refutation: practices of *elenchus* in ancient Greek dialectic, and the formulation of counterexamples to proposed analyses of concepts, in particular as illustrated by the Gettier problem in the second half of the twentieth century. I have argued that, while it might seem that an *elenchus* is not a desirable model for philosophical refutation on account of being *ad hominem*, if suitably approached an *elenchus* is in fact an outstanding method for questioning the obvious. I have emphasized its therapeutic (in the Socratic version) and cooperative (in the Aristotelian version) components. By contrast, approaches to refutation primarily focused on the formulation of counterexamples, while potentially fruitful, also pose the risk of producing philosophical reflections that are alienated from the relevant human experiences. Philosophical argumentation of this kind can become an arm’s race where claims and counterexamples become increasingly convoluted and fanciful. The debates on the Gettier problem in the second half of the twentieth century (and beyond) seem to be a good illustration of this dynamic.

But this does not mean that the counterexample approach to philosophical refutation should be completely abandoned. Rather, what is required are clearer guidelines on *how* to engage in refutation by counterexample responsibly and fruitfully. I have argued that Lakatos’ *Proofs and Refutations*, while being ostensibly about mathematics, provides interesting insights also for philosophical argumentation. But while Lakatos speaks against monster-barring in mathematics, I have suggested that monster-barring should at least sometimes be deployed in philosophy—that is, if philosophical inquiry is to remain suitably connected to human experiences (while still questioning the obvious).

## Footnotes

1 To probe whether this is a trivial or interesting question, I ran a poll on Twitter (admittedly, not a very rigorous method of evidence gathering!), which received just under 100 votes. To my surprise, the votes on the two options—philosophical argumentation does not differ substantially from argumentation in other spheres vs. there are substantial differences—were almost evenly distributed. (I had expected that there would be many more votes for the second option.).

2 Notice however that Popper uses the term ‘refutations’ to refer to the (empirical) falsification of conjectures in the empirical sciences (Popper 1989). Incidentally, the title of Lakatos’ book *Proofs and Refutations*, to be extensively discussed below, is an explicit reference to Popper’s famous essay, ‘Conjectures and Refutations’ (published in the eponymous book).

3 This section draws substantially on the analysis of dialectical refutations presented in Dutilh Novaes (2020a, b).

4 There are a number of detailed reconstructions of the basic features of this practice in the literature (Castelnérac and Marion 2009, 2013; Fink 2012). Scholars disagree on how best to understand the concept of dialectic, even in the restricted context of ancient Greek thought (Duncombe and Dutilh Novaes 2016). It is important to bear in mind that Socratic dialectic, as depicted in Plato’s early dialogues, is different from the notion of dialectic that emerges from Plato’s later dialogues. Here we focus on Socratic dialectic specifically.

5 The battle metaphor for a dialectical encounter is also present in the *Philebus*.

6 However, we should not take Plato’s account as telling the whole story about what *elenchi* were for his immediate predecessors and his contemporaries (Lesher 2002). More likely, the term was used to cover related but nevertheless diverse argumentative strategies (Castelnérac and Marion 2009).

7 There is an interesting discussion in the literature on whether philosophical argumentation is overly adversarial due to the centrality of refutation in these practices; this is a concern voiced in particular by a number of feminist thinkers (Moulton 1983; Hundleby 2021). Elsewhere (Dutilh Novaes 2021), I’ve argued that adversariality in argumentation is not bad per se, but it needs to be tempered so as to be proportional to what is required by the situation. See also Aikin (2011) and Kidd (2020) who present similar ideas, and Callard (2019) on the value of ‘fighting’ in philosophy.

8 I owe the point on the influence of logic and mathematics within this philosophical tradition to an anonymous referee. To be sure, counterexamples are also important in the context of Socratic dialectic (Matthews 2009), in particular if they reflect common opinion (*endoxa*). But they are one element within broader strategies of refutation, whereas in the twentieth century they become the primary focus of refutations. (In the ancient Greek tradition, close connections between philosophical and mathematical argumentative practices were also in place (Dutilh Novaes 2020b).).

9 Indeed, in Gettier (1963), some logical machinery is deployed, in particular to formulate the principle of epistemic closure, which is crucial for his main argument (I owe this point to an anonymous referee).

10 “It became something of a convenient fiction to suppose that this analysis was widely accepted throughout much of the in history of philosophy. In fact, however, the JTB analysis was first articulated in the twentieth century by its attackers.” (Ichikawa and Steup 2018) (Section 1.) (See also Dutant 2015).). Indeed, it is rather common for philosophers to formulate a view that hasn’t really explicitly been defended by anyone, to claim that it is the ‘traditional’ view, and then to go on to ‘refute’ the view with a devastating counterexample or some other killing objection. (If this approach strikes the reader as somewhat dissatisfying, I concur.).

11 This assumption was questioned by so-called experimental philosophers in the early 2000’s, and an interesting discussion on empirical testing of Gettier cases has emerged (Turri 2016). See also Weinberg (2016) on the role of intuitions in philosophical inquiry more generally.

12 Interestingly, Humanist thinkers in the sixteenth century levelled this very criticism against scholasticism (Nauta 2009). Also telling is the proverbial discussion on ‘how many angels can dance on the head of a pin?’, which was initially formulated as a critique of scholasticism, angelology in particular.

13 Indeed, it has been argued that the dynamics described in *Proofs and Refutations* can be observed in only a handful of actual case studies from the history of mathematics (Koetsier 1991), or in recent mathematical developments (Tanswell 2017) (section 3.3).

14 Notice though that the historical accuracy of this rational reconstruction has been contested (Musgrave and Pigden 2016) (section 3.1).
